# Regional reduction of high-energy phosphorus metabolites characterizes the cerebellar subtype of multiple system atrophy

**DOI:** 10.1038/s41531-026-01537-y

**Published:** 2026-08-20

**Authors:** Jannik Prasuhn, Christina Bodemann, Britt Ebeling, Katharina Reuther, Sinja S. Großer, Jan Uter, Julia Henkel, Henrike Hanssen, Norbert Brüggemann

**Affiliations:** 1 Section of Movement Disorders, Department of Neurology, University Medical Center Schleswig-Holstein, Lübeck, Germany; 2 Institute of Neurogenetics, University of Lübeck, Lübeck, Germany; 3 Center for Brain, Behavior, and Metabolism, University of Lübeck, Lübeck, Germany; 4 Division of MR Research, Department of Radiology, Johns Hopkins University School of Medicine, Baltimore, MD USA; 5 F.M. Kirby Research Center for Functional Brain Imaging, Hugo W. Moser Research Institute at Kennedy Krieger, Baltimore, MD USA

**Keywords:** Diseases, Neurology, Neuroscience

## Abstract

Multiple system atrophy (MSA) is a severe neurodegenerative disorder with various underlying pathophysiological features. Mitochondrial dysfunction has been implied as a viable treatment target in patients with MSA. Yet, there is a lack of in-vivo studies examining regional metabolic differences between the Parkinsonian (MSAp) and the cerebellar (MSAc) subtype of MSA. Twenty-four patients with MSA (12 patients with MSAp and 12 patients with MSAc), 24 patients with Parkinson’s disease (PD), and 24 age- and sex-matched healthy controls (HCs) underwent clinical evaluations and multimodal neuroimaging, including ^31^P-MRSI targeting the basal ganglia and the cerebellum. Ratios of high-energy phosphorus-containing metabolites (HEPs) were compared between groups. Only patients with MSAc showed decreased HEP levels in the cerebellum. Conversely, no differences in basal ganglia HEP levels appeared between both MSA subtypes, patients with PD, or HCs. Our findings provide preliminary in-vivo evidence for regionally detectable bioenergetic alterations in the cerebellum of patients with MSAc, while basal ganglia results, particularly in MSAp, require cautious interpretation because of the spatial-resolution limits of the present ^31^P-MRSI approach.

## Introduction

Multiple system atrophy (MSA) is an adult-onset neurodegenerative disorder that manifests primarily with two subtypes: parkinsonian (MSAp) and cerebellar (MSAc), each defined by specific patterns of neurodegeneration and respective clinical presentations^1,2^. In our current understanding, α-synuclein (aSyn) accumulation in glial cells, particularly oligodendrocytes, can be considered a pathophysiological hallmark in patients with MSA (PwMSA)^3^. The cellular and metabolic downstream effects of aSyn deposition and aggregation in MSA remain subject of intense investigation^4–6^. Beyond the immediate cytotoxic effects, aggregated aSyn has widespread implications on cellular metabolism, particularly oxidative phosphorylation via the mitochondria^7,8^. Here, the aSyn protein interferes with mitochondrial proteins of the outer mitochondrial membrane, subsequently causing bioenergetic disturbances^5^. The pivotal role of mitochondrial metabolism in mammalian cells implies that any perturbation in its function can have deleterious effects on neural homeostasis and subsequently lead to neurodegenerative processes^5^. Emerging evidence suggests that bioenergetic profiles may differ between MSA subtypes, hinting at disparate pathophysiological mechanisms in MSAp and MSAc. For example, variations in the *COQ2* gene (relevant for coenzyme Q_10_, CoQ_10_, biosynthesis, and mitochondrial homeostasis) appears to increase the susceptibility predominantly for the MSAc subtype^9,10^. Besides the in-vitro scientific evidence on the role of glial aSyn-deposition and -aggregation and the subsequent impact on mitochondrial functioning, in-vivo evidence, however, is lacking. ^31^Phosphorus magnetic resonance spectroscopy imaging (^31^P-MRSI), a non-invasive imaging modality, offers an avenue to explore these differences non-invasively and in vivo^11^. The comparison of region-specific metabolite profiles between patients with MSAp (PwMSAp), patients with MSAc (PwMSAc), and age- and sex-matched healthy controls (HCs) could provide in-vivo evidence for bioenergetic disturbances and could elucidate the nuanced metabolic shifts inherent to each MSA subtype. This information would not only enhance our current understanding of the underlying disease mechanisms but could also help to evaluate bioenergetic disturbances due to mitochondrial dysfunction as a potential treatment target. Mitochondrial dysfunction has been a beacon for therapeutic exploration in neurodegenerative diseases mainly for two reasons:

First, understanding these imbalances in detail might offer diagnostic markers for early detection and intervention. Both are crucial prerequisites for the development of disease-modifying treatments^12^.

Secondly, identifying and correcting bioenergetic disturbances might mitigate some downstream effects of such imbalances, such as excessive oxidative stress^13^.

There have been some preliminary attempts to leverage these insights to conceptualize clinical trials^14^. For instance, compounds that enhance mitochondrial function have been proposed as potential therapeutic agents for MSA (e.g., high-dosage treatments with CoQ_10_)^15,16^. However, the efficacy of such therapies remains a topic of ongoing debate, and the results, though promising in some studies, have been inconsistent^17^. Our current study sought to investigate whether ^31^P-MRSI can detect regional differences in high-energy phosphorus metabolite ratios between MSA subtypes in vivo. Given the exploratory nature and technical constraints of this approach, particularly the limited spatial specificity of ^31^P-MRSI at 3T, we aimed to assess whether subtype-associated regional bioenergetic alterations can be detected rather than to establish a diagnostic biomarker at this stage.

## Results

### Demographics and clinical data

We recruited twelve PwMSAp (six male, six female; age: 62.6 ± 8.6 years), twelve PwMSAc (seven male, five female; age: 61.5 ± 5.5 years), 24 PwPD (twelve male, twelve female; age: 64.0 ± 6.2 years, which have been previously published)^18^, and 24 HCs (11 male, 13 female; age: 65.6 ± 5.7 years). Groups were well matched for age (*p* = 0.553) and sex distribution (*p* = 0.919). The PwMSA were in a mid-stage disease phase, with a mean disease duration of 39.2 ± 17.5 months for PwMSAp and 50.1 ± 28.0 months for PwMSAc, while PwPD had a mean duration of 60.4 ± 22.7 months (*p* = 0.052 across groups). PwMSAp and PwMSAc showed a comparable motor-symptom burden, with UMSARS-II scores of 20.3 ± 7.0 and 15.0 ± 10.7, respectively (*p* = 0.985), and a median UMSARS-IV score of 3 (range: 1–4) in both subgroups (*p* = 0.415). Phenotype-specific scales further characterized disease expression: PwMSAp had a mean MDS-UPDRS-III score of 34.0 ± 11.3, PwPD 28.6 ± 9.8, with significant differences across groups (*p* = 0.033), while PwMSAc had a mean SARA score of 8.5 ± 2.1. With respect to dopaminergic treatment, PwMSAp had a mean LEDD of 420 ± 210 mg/day, PwMSAc of 310 ± 185 mg/day, and PwPD of 520 ± 260 mg/day (*p* = 0.041). The proportion of patients receiving levodopa was 75% (9/12) in PwMSAp, 58% (7/12) in PwMSAc, and 83% (20/24) in PwPD (*p* = 0.038). When considering any form of dopaminergic medication, the respective proportions increased to 83% (10/12), 67% (8/12), and 92% (22/24), with no such treatment in HCs (*p* < 0.001). All demographic and clinical data are summarized in Table 1.

**Table 1:** Baseline demographics, clinical characteristics, and neuroimaging-derived measures

	PwMSAp (*n* = 12)	PwMSAc (*n* = 12)	PwPD (*n* = 24)	HCs (*n* = 24)	*p*-value
	Demographics				
Age [years]	62.6 ± 8.6	61.5 ± 5.5	64.0 ± 6.2	65.6 ± 5.7	0.553
Sex [m/f]	6/6	7/5	12/12	11/13	0.919^a^
	Clinical characteristics				
Disease duration [months]	39.2 ± 17.5	50.1 ± 28.0	60.4 ± 22.7	N/A	0.052
UMSARS-II	19.6 ± 7.7	19.0 ± 10.5	N/A	N/A	0.985
UMSARS-IV	3 (1; 4)^c^	3 (1; 4)^c^	N/A	N/A	0.415^b^
MDS-UPDRS-III	34.0 ± 11.3	N/A	28.6 ± 9.8	N/A	0.033
SARA	N/A	8.5 ± 2.1	N/A	N/A	N/A
LEDD [mg/d]	420 ± 210	310 ± 185	520 ± 260	N/A	0.041
Patients on levodopa [%]	75	58	100	N/A	0.038
	Neuroimaging (T1-weighted neuroimaging-derived volumetry)				
Gray matter (total) [% TIV]	52.6 ± 0.96	51.2 ± 1.36	50.8 ± 3.41	51.2 ± 3.32	0.084
White matter (total) [% TIV]	29.8 ± 1.61	29.6 ± 2.45	29.7 ± 4.28	30.2 ± 4.35	0.977
Cerebrum [% TIV]	73.4 ± 1.73	73.1 ± 3.43	72.1 ± 3.29	72.7 ± 3.34	0.906
Cerebellum [% TIV]	8.7 ± 0.81	6.9 ± 0.84	8.3 ± 0.81	8.5 ± 0.77	<0.001
Pallidum [% TIV]	0.13 ± 0.022	0.17 ± 0.041	0.15 ± 0.046	0.16 ± 0.049	0.008
Caudate [% TIV]	0.33 ± 0.047	0.37 ± 0.040	0.36 ± 0.012	0.37 ± 0.011	<0.001
Putamen [% TIV]	0.32 ± 0.055	0.50 ± 0.072	0.49 ± 0.009	0.50 ± 0.010	<0.001

Values are reported as mean ± standard deviation.

Group differences were calculated using ANOVA analyses if not stated otherwise.

*f* female, *HCs* healthy controls, *LEDD* levodopa equivalent daily dose, *m* male, *MDS-UPDRS-III* Movement Disorders Society Unified Parkinson’s Disease Rating Scale, subscore III, *N/A* not applicable, *PwMSAc* patients with multiple system atrophy, cerebellar subtype, *PwMSAp* patients with multiple system atrophy, Parkinsonian subtype, *PwPD* patients with Parkinson’s disease, *SARA* Scale for the Assessment and Rating of Ataxia, *TIV* total intracranial volume, *UMSARS* Unified Multiple System Atrophy rating scale.

^a^Chi-square and ^b^Mann–Whitney U test have been employed.

^c^These values are reported as median (minimum; maximum).

### T1-weighted neuroimaging-derived volumetry reveals MSA subtype-specific atrophy patterns

Group comparisons of T1-weighted volumetric measures are summarized in Table 1. For global measures, no systematic differences were found between PwMSA and HCs. Total gray matter was slightly higher in PwMSAp (+2.8%; 52.6 ± 0.96% TIV) and unchanged in PwMSAc (+0.0%; 51.2 ± 1.36% TIV) compared to HCs (51.2 ± 3.32% TIV; *p* = 0.084). Total white matter was comparable between PwMSAp (–1.3%; 29.8 ± 1.61% TIV) and PwMSAc (–2.0%; 29.6 ± 2.45% TIV) relative to HCs (30.2 ± 4.35% TIV; *p* = 0.977). Cerebral volume was also comparable between both PwMSAp (+1.0%; 73.4 ± 1.73% TIV) and PwMSAc (+0.6%; 73.1 ± 3.43% TIV) compared to HCs (72.7 ± 3.34% TIV; *p* = 0.906).

Regionally, PwMSAp exhibited marked volume reductions in the pallidum (–18.8%; 0.13 ± 0.022% TIV vs. 0.16 ± 0.049% TIV; *p* = 0.008), caudate (–10.8%; 0.33 ± 0.047% TIV vs. 0.37 ± 0.011% TIV; *p* < 0.001), and putamen (–36.0%; 0.32 ± 0.055% TIV vs. 0.50 ± 0.010% TIV; *p* < 0.001). These reductions were not observed in PwMSAc, who instead showed a pronounced cerebellar atrophy (–18.8%; 6.9 ± 0.84% TIV vs. 8.5 ± 0.77% TIV; *p* < 0.001). In PwPD, no evidence of structural atrophy was found compared to HCs, with volumetric values being nearly identical across all regions (all *p* > 0.05).

### ^31^P-MRSI-derived metabolite ratios indicate a reduction of HEPs in the cerebellum of PwMSAc

First, we performed ANCOVA analyses for the basal ganglia HEP levels. Here, all three models passed visual inspection of Q-Q-plots and Levene’s test for homogeneity of variances: (ATP + PCr)/iP: *p* = 0.868, ATP/iP: *p* = 0.207, PCr/iP: *p* = 0.563. None of the models detected any statistically significant differences between PwMSAp, PwMSAc, and HCs for the basal ganglia HEP levels (corrected for age, sex, and TIV): (ATP + PCr)/iP: F(2, 42) = 0.55 *p* = 0.579, ATP/iP: F(2, 42) = 1.66 *p* = 0.202, PCr/iP: F(2,42) = 0.23 *p* = 0.792.

In the following, we performed ANCOVA analyses for the cerebellar HEP levels. Again, all three models passed visual inspection of Q-Q-plots and Levene’s test for homogeneity of variances: (ATP + PCr)/iP: *p* = 0.499, ATP/iP: *p* = 0.479, PCr/iP: *p* = 0.341. The ANCOVA model was statistically significant due to lower (25.6%) cerebellar (ATP + PCr)/iP levels (F(2, 42) = 8.60, *p* ≤ 0.001) in PwMSAc compared to HCs. There was no observable influence of age (*p* = 0.184), sex (*p* = 0.766), or TIV (*p* = 0.237) on this finding. Post-hoc analyses revealed that these results were driven by the group differences between PmMSAc vs. HCs (t(42) = 6.28, *p*_Tukey_ ≤ 0.001) and PwMSAc vs. PwMSAp (t(42) = 6.08, *p*_Tukey_ ≤ 0.001). No differences between PwMSAp and HCs were detectable for cerebellar HEP levels (t(42) = 0.74, *p* = 0.859).

Similar group differences were seen for cerebellar ATP/iP levels (F(2, 42) = 7.98, *p* = 0.001) with 26.1% lower levels in PwMSAc compared to HCs. For ATP/iP, no influences of age (*p* = 0.508), sex (*p* = 0.984), and TIV (*p* = 0.685) were seen. Post-hoc analyses revealed that these differences were due to the group comparison of PmMSAc vs. HCs (t(42) = 5.28, *p*_Tukey_ = 0.002) and PwMSAc vs. PwMSAp (t(42) = 4.69, *p*_Tukey_ = 0.005). No differences were detectable between PwMSAp and HCs.

Group differences were also present for cerebellar PCr/iP levels (F(2, 42) = 8.74, *p* ≤ 0.001) with 22.0% lower levels in PwMSAc compared to HCs. For PCr/iP, no influences of age (*p* = 0.188), sex (*p* = 0.749), and TIV (*p* = 0.230) were seen. Post-hoc analyses revealed that these differences were consistently caused by the comparison PmMSAc vs. HCs (t(42) = 5.17, *p*_Tukey_ = 0.002) and PwMSAc vs. PwMSAp (t(42) = 5.01, *p*_Tukey_ = 0.003). No differences were detectable between PwMSAp and HCs.

**Fig. 1:**
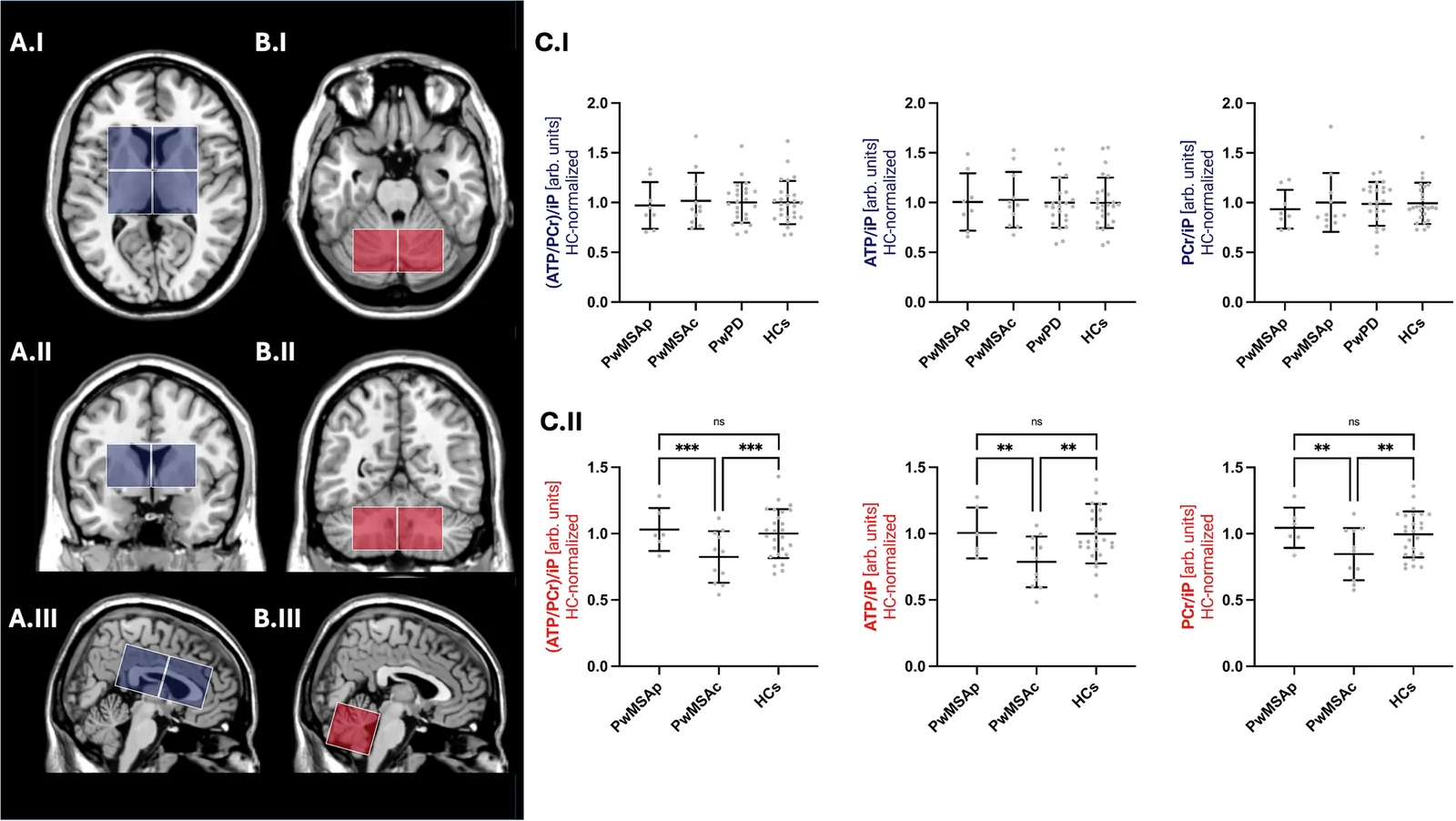
^31^P-MRSI voxel placement and descriptive statistics of the bioenergetic state in PwMSAp, PwMSAc, PwPD, and HCs. In Panel **A**.I, we display the basal ganglia voxel placement (blue boxes) for the ^31^P-MRSI chemical shift imaging grid/voxel in the axial (**A**.I), coronal (**A**.II), and sagggital (**A**.III) planes. Panel **B** depicts the voxel placement (red boxes) in the cerebellar hemispheres in the axial (**B**.I), coronal **B**.II), and sagittal (**B**.III) planes. All four basal ganglia and the two cerebellar voxels were averaged to calculate the metabolite ratios subsequently. In panels **C**.I and **C**.II, we highlight the individual data points and descriptive statistics (mean ± SD) for three metabolite ratios derived from both neuroanatomical regions (the basal ganglia, highlighted in blue, panel **C**.I, and the cerebellum, highlighted in red, panel **C**.II). To enhance the readability, we expressed all parameters in relation to the mean of the HCs group (HC-normalized). Significant results from the post-hoc analyses are highlighted with asterisks for illustrative purposes (**p ≤0.01, ***p ≤0.001). ^31^P-MRSI ^31^phosphorus magnetic resonance spectroscopy imaging, arb. units arbitrary units, ATP adenosine triphosphate, HCs healthy controls, iP inorganic phosphate, ns non-significant, PCr phosphocreatine, PwMSAc patients with multiple system atrophy, cerebellar type, PwMSAp patients with multiple system atrophy, Parkinsonian type, PwPD patients with Parkinson’s disease.

Importantly, in PwPD we likewise did not observe any differences in basal ganglia HEP levels compared to either PwMSA or HCs in medication OFF state. Results are also reported in Fig. 1. We repeated all the above-stated analyses without using iP as a denominator to calculate metabolite ratios. We did not detect any mentionable differences with respect to our already reported analyses (data not shown here).

An exploratory sample-size estimation based on the observed ANCOVA effects for basal-ganglia HEP ratios indicated that substantially larger cohorts would be required to detect the small effects observed in this region with adequate statistical power. The observed partial η² values were 0.026 for (ATP + PCr)/iP, 0.073 for ATP/iP, and 0.011 for PCr/iP, corresponding to Cohen’s f values of 0.162, 0.281, and 0.105, respectively. Assuming α = 0.05, 80% power, and three groups, these effects would require approximately 371, 125, and 883 participants in total, respectively. Therefore, the absence of significant basal-ganglia HEP differences in the present cohort should be interpreted cautiously.

### ^31^P-MRSI-derived metabolite ratios are neither cofounded by neuroanatomical differences nor reveal any association with clinical data

As we have only seen group differences in HEP levels in the cerebellum of PwMSAc, we focussed our subsequent analyses on this subgroup and neuroanatomical structure. Here, none of the three investigated metabolite ratios revealed any significant correlation with the cerebellar volume in PwMSAc. In addition, none of the three metabolite ratios was significantly correlated with the age, disease duration, UMSARS subscore II, IV, or the SARA score of PwMSAc. Likewise, in the PwPD cohort, we did not observe any significant results for any of the tested correlations.

## Discussion

This study examined whether ^31^P-MRSI can detect regional differences in high-energy phosphorus metabolite ratios between MSA subtypes in vivo. The most robust finding was a reduction of cerebellar HEP ratios in patients with MSA-C. In contrast, no basal ganglia differences were detectable in MSA-P with the present protocol; however, this result must be interpreted cautiously because the applied VOI was large relative to the anatomical structures of interest and inevitably included surrounding tissue. This divergence not only lends weight to the notion that the two MSA subtypes have underlying differences in their pathophysiological trajectories but also emphasizes the heterogeneity of the impact of aSyn deposition and aggregation on neuronal and glial cells in different brain regions^3,6^. Our study underscores the necessity of a more nuanced understanding of the pathophysiological role of aSyn in the context of metabolic changes in neuroanatomically defined structures. Previous studies investigated the role of CoQ_10_ levels as a surrogate marker for mitochondrial homeostasis in serum and cerebrospinal fluid levels in PwMSA^19,20^. However, distinct differences in CoQ_10_ levels between PwMSAp and PwMSAc were not detectable^19,20^. These findings contrast with genetic studies demonstrating that variants in the *COQ2* gene increase the susceptibility for the MSAc subtype and *post-mortem* analyses showing a decrease of CoQ_10_ levels in the cerebellum of PwMSAc^9,10,21,22^. These conflicting results point toward more nuanced considerations of neuroanatomical structures in MSA subtypes. Interestingly, reduced *COQ2* expression levels have been reported to be associated with lowered ATP levels in PwMSA-derived brain tissue^23^. In summary, these reports reveal a tendency for bioenergetic changes to be more specific for PwMSAc, which fits our in-vivo observations. For PwMSAp, this leads us to consider other modulating factors or co-pathologies that might be at play in determining the absent (or non-detectable) bioenergetic changes in the basal ganglia. In our study, the observation of structural atrophy in the basal ganglia of PwMSAp without corresponding changes in HEP levels raises intriguing possibilities about the underlying mechanisms of this particular subtype: One explanation could be the presence of compensatory metabolic processes within the basal ganglia. Such compensatory mechanisms might be maintaining HEP levels, effectively masking the mitochondrial dysfunction that one would expect to accompany structural degeneration. Additionally, the stage of disease at the time of our measurements might also play a crucial role. If mitochondrial dysfunction in PwMSAp is a stage-dependent phenomenon, the patients in our study might not have reached the point where these changes are bioenergetically apparent. Furthermore, the potential pathophysiological heterogeneity in MSA-P could suggest that mitochondrial dysfunction might not be a predominant feature across all individuals (as, e.g., discussed in the context of the genetic underpinnings of the MSA-C subtype)^10,23^. Interestingly, cerebellar HEP ratios did not correlate with cerebellar volume in MSAc. This apparent decoupling may have several explanations. From a biological perspective, bioenergetic impairment could precede overt tissue loss or reflect mitochondrial dysfunction in surviving neurons, glial cells, or synaptic compartments rather than simply mirroring neuronal loss. Thus, reduced HEP ratios may capture a functional metabolic state that is not linearly related to macroscopic atrophy. Alternatively, this negative correlation finding may partly reflect technical limitations. The relatively large ^31^P-MRSI voxels provide a regionally averaged metabolic signal and are not spatially matched to fine-grained anatomical volume measures. Partial-volume effects and limited anatomical specificity may therefore reduce sensitivity to detect associations between local tissue loss and local metabolic impairment. Longitudinal studies with higher-resolution ^31^P-MRSI and repeated structural imaging will be required to determine whether bioenergetic alterations precede, accompany, or follow cerebellar atrophy in MSAc. Another important aspect relates to the relatively large VOI applied for ^31^P-MRSI, which encompasses both gray and white matter structures. This lack of spatial specificity towards the striatum, the primary site of pathology, likely reduces the SNR of the metabolic signal and may therefore attenuate the sensitivity to detect subtle region-specific bioenergetic changes. Taken together, this complex interplay of technical and biological factors underscores the intricate and multifaceted nature of neurodegenerative diseases, highlighting the need for a broader spectrum of pathophysiology-orientated neuroimaging to unravel the specific contributions and interactions of these various elements in future studies. This more nuanced understanding can be considered a prerequisite for patient stratification (between the different MSA subtypes or within the PwMSAc group) and therapeutic monitoring. Here, the pronounced bioenergetic impairment in the cerebellum could indicate that therapies focused on enhancing mitochondrial ATP production could be particularly beneficial in a predefined subset of PwMSAc. By leveraging these metabolic markers, clinicians might be equipped to make more informed prognostic assessments and tailored treatment plans, as MRSI-based assessments of the bioenergetic state are particularly suitable for the longitudinal evaluation of patients. Previously, we have demonstrated the suitability of this approach in a case series of patients with hereditary ataxia caused by mutations implicated in CoQ_10_ biosynthesis^24^.

While our study provides compelling insights, certain limitations need to be noted.

The small sample size represents an important limitation, particularly in view of the clinical and biological heterogeneity of MSA. Our exploratory sample-size estimation showed that the observed basal-ganglia effects were small and would require substantially larger cohorts to be detected with adequate statistical power. Therefore, the absence of detectable basal-ganglia HEP differences in MSAp may reflect limited statistical power and should not be interpreted as evidence for preserved bioenergetic function. Rather, these findings should be regarded as preliminary pilot observations within the anatomical and statistical limits of the present study.

Additionally, ^31^P-MRSI has inherent resolution and sensitivity limits, particularly at clinically available field strengths^11^. The VOIs used in the present study were therefore comparatively large, reflecting the necessary trade-off between spatial resolution, signal-to-noise ratio, metabolite detectability, and clinically feasible scan time. This limitation is especially relevant for the basal ganglia, where the selected voxels inevitably included surrounding tissue and therefore did not permit a strictly striatal or pallidal assessment of HEP levels. In the cerebellum, the VOI provided a broader estimate of cerebellar bioenergetic state, but likewise did not allow subregional analyses of specific cerebellar lobules or nuclei. Future studies using higher-field MRI, optimized ^31^P-coils, advanced acquisition strategies, and improved reconstruction methods will be needed to further narrow VOI placement and to determine whether more focal HEP alterations are detectable in specific basal ganglia or cerebellar subregions. Such developments will be particularly relevant for studying early-stage MSA, in which region-specific metabolic abnormalities may precede or occur independently of pronounced structural atrophy. However, our study allowed the measurement of HEP levels in two distinct neuroanatomical regions, something that frequently used surface head coils can not achieve. These findings support the presence of subtype-associated regional bioenergetic alterations in MSA, most clearly involving the cerebellum in MSAc. Larger, longitudinal, and technically optimized studies are required to determine whether ^31^P-MRSI-derived HEP measures can contribute to early-stage characterization, patient stratification, or treatment monitoring in MSA. An additional limitation is that the present ^31^P-MRSI protocol did not include a dedicated midbrain or *substantia nigra* VOI. This is particularly relevant for MSAp, given its striatonigral pathology and recent evidence that midbrain energy-homeostasis measures may be informative in parkinsonian syndromes. Therefore, the absence of detectable HEP reductions in our basal-ganglia VOI should not be interpreted as evidence against bioenergetic impairment in MSAp, but only as a negative finding within the sampled basal-ganglia region. Dedicated midbrain ^31^P-MRSI, however, remains technically challenging at 3T because of the small size and deep location of the *substantia nigra*, partial-volume effects, and demanding shimming conditions. Future studies using optimized acquisition strategies, higher field strengths, and improved shimming may be able to clarify whether focal mesencephalic bioenergetic alterations are present in MSAp.

A further limitation is that the present study does not establish diagnostic performance. We did not test classification accuracy, sensitivity, specificity, or predictive value, and the sample size was not designed for diagnostic model development. Therefore, the observed cerebellar HEP reduction in MSAc should be interpreted as a group-level pathophysiological finding rather than as a clinically applicable diagnostic marker. Similarly, the absence of detectable basal ganglia HEP differences in MSAp cannot be interpreted as evidence against bioenergetic involvement of basal ganglia structures, because the spatial resolution and partial-volume characteristics of the current ^31^P-MRSI protocol may have limited sensitivity to focal striatal or pallidal abnormalities. If replicated in larger and longitudinal cohorts, such regional bioenergetic measures may contribute to future biomarker development for patient stratification or therapeutic monitoring. However, the present study should be regarded as an exploratory step toward this goal and does not establish diagnostic utility. Complementary neuroimaging techniques could foster a more nuanced evaluation of the complex concept of mitochondrial dysfunction in these patient cohorts (e.g., approaches suitable for mapping the presence of oxidative stress)^11^. Additionally, longitudinal studies could lead to a refined understanding of the temporal dynamics of mitochondrial dysfunction in MSA subtypes. In particular, evaluating patients in a prodromal state could open up a window of opportunity for disease-modifying treatments, but identifying patients in this disease stage is inherently difficult to achieve. In this study, PwMSA have been included when pronounced atrophy was already present, additionally impacting the role of our bioenergetic findings. However, no correlation between the degree of atrophy and the bioenergetic state has been observed, which aligns well with our previous reports^18,24,25^.

In summary, our study highlights the in-vivo metabolic differences between MSA subtypes. These findings accentuate the distinct pathophysiological trajectories of PwMSAp and PwMSAc and present potential avenues for targeted diagnostics and therapeutics. Integrating pathophysiology-targeted neuroimaging will undoubtedly pave the way for more effective, precision-based interventions in patients with neurodegenerative disorders.

## Methods

### Recruitment and clinical assessments

The present study has been performed following the revised version of the Declaration of Helsinki and received prior approval from the institutional review board of the University of Lübeck (AZ 18-196). All participants gave their informed written consent before any study-related activities were performed.

We have enrolled PwMSA if the highest level of clinical diagnostic certainty has been met, as defined by the term *clinically established* MSA based on the revised diagnostic criteria of the Movement Disorders Society (MDS). The same approach was applied for PwPD, where only patients fulfilling the MDS clinical diagnostic criteria for *clinically established* PD were included^26,27^. We assessed demographic data, the medical history, the current medication state, and potential exclusion criteria (e.g., the presence of any other neurological comorbidity). To account for potential differences in the respective treatment regimes we calculated the levodopa-equivalent daily dosage (LEDD) across the reported groups. In addition, we also considered the proportion of patients receiving levodopa and, more generally, any form of dopaminergic medication, expressed as percentages within each group. Study participants were recruited via our in- and outpatient clinics. Certified movement disorders specialists performed a standardized neurological examination following the Unified Multiple System Atrophy Rating Scale (UMSARS), the MDS-Unified Parkinson Disease Rating Scale (MDS-UPDRS, for PwMSAp, and PwPD), and the Scale for the Assessment and Rating of Ataxia (SARA, for PwMSAc)^28–30^.

### Neuroimaging acquisition

We performed multimodal neuroimaging sessions at the *Center for Brain, Behavior, and Metabolism* imaging core facility utilizing a 3T Siemens MAGNETOM Skyra scanner. Structural brain images were acquired with a standard 3D T1-weighted MP-RAGE sequence with the following protocol parameter: GRAPPA: factor 2; TR: 1900 ms; TE: 2.44 ms; TI: 900 ms; flip angle: 9°; 1 × 1 × 1 mm^3^ resolution; 256 × 256 × 256 mm^3^ field of view; total scan time: 4 min and 33 s.

The ^31^P-MRSI was performed using a dual-tuned quadrature ^1^H/^31^P head coil (RAPID Biomedical) via a 3D chemical shift imaging (CSI) sequence following the subsequent protocol parameters: TR: 2000 ms; TE: 2.3 ms; flip angle: 50°; 30 × 30 × 30 mm^3^ resolution; 240 × 240 × 240 mm^3^ field of view; 8 × 8 × 8 matrix size; 6-fold weighted averaging; Hamming filtering; wideband alternating-phase low-power technique for zero-residual splitting decoupling (WALTZ-4); total scan time: 8 min and 4 s. The CSI grid placement and voxel selection have been highlighted in Fig. 1. Here, specific neuroanatomical landmarks (ventricular anterior hons and the frontal border of the corpus callosum for the basal ganglia, the third ventricle, and the central line between the cerebellar hemispheres for the cerebellum, Fig. 1) have been used for CSI grid placement. Before the datasets were considered for further analyses, the presence of structural lesions was excluded by senior neuroradiologists.

### Neuroimaging postprocessing and analyses

Metabolite spectra were quantified on a dedicated Siemens workstation (SynGo MR ER11 and VH22B_Sl19P26_CSI03 software package). For the following quantification, we used known nuclear magnetic resonance attributes of phosphorus-containing metabolites (e.g., such as ATP)^31^. The analysis incorporated the following steps: Fourier transformation utilizing a Hanning filter (200-ms width, centered at 0 ms) and zero padding (2048); adjustments for voxel-wise frequency-shift (setting the PCr signal at 0 Hz); automated phase correction using a constant phase only, with peak positions of ATP, phosphocreatine (PCr), and inorganic phosphate (Pi); a 5th order polynomial baseline adjustment; and non-linear Gaussian curve fitting (taking into account J-coupling constants of α-, β-, and γATP peaks).

Before subsequent analysis, metabolite levels were averaged for the basal ganglia (four neighboring voxels) and the cerebellum (two neighboring voxels, Fig. 1). In this report, we present high-energy phosphorus-containing metabolites (HEPs) as ratios normalized to iP (to additionally account for intraindividual differences, e.g., in the dietary intake of phosphorus-containing metabolites). Here, we used the αATP peaks for subsequent quantification of the total ATP levels. ATP and PCr form a highly dynamic equilibrium in depicting the overall bioenergetic state. We, therefore, considered (ATP + PCr)/iP the primary neuroimaging outcome measure to compare the bioenergetic state between PwMSAp, PwMSAc, PwPD, and HCs. To exclude the potential influence of using metabolite ratios, we additionally re-analyzed our data without using iP as a denominator.

In addition, we performed volumetric analyses derived from T1-weighted images to compare regional atrophy patterns as potential confounding variables for the metabolite ratios. Volumetric measures of distinct neuroanatomical regions of interest (ROIs) were extracted from T1-weighted images via the *AssemblyNet* processing pipeline. Here, a large ensemble of convolution neural networks was used to segment T1-weighted images according to the *BrainColor* atlas^32^. Total gray matter volumes (uncorrected for the total intracranial volume, TIV) were used as correlation targets for ^31^P-MRSI-derived metabolite ratios. In addition, we report gray matter volumes normalized to TIV for group comparisons.

### Statistics

For our primary hypothesis, we performed a two-tailed analysis of covariance (ANCOVA) with the group assignment (PwMSAp, PwMSAc, PwPD, and HCs) as the independent variable and the (ATP + PCr)/iP as the dependent variable. Age, sex, and TIV were used as covariates. We performed the same analysis with ATP/iP and PCr/iP as dependent variables as secondary outcome measures. For the cohort of PwPD, cerebellar values were not available; therefore, comparisons in this group were restricted to the basal ganglia against PwMSA and HCs. To identify associations between our neuroimaging measures and between our neuroimaging measures and clinical data, we performed Pearson’s correlations. In this report, we considered the correlative analyses exploratorily, and all results are reported uncorrected. All above-stated analyses were performed only when necessary assumptions (e.g., the normal distribution of variables) were met. Post-hoc analyses were performed with Tukey correction for multiple comparisons. Descriptive statistics are reported as mean ± standard deviations (SD) if not stated otherwise. Statistical analyses were done in Jamovi (version 2.3.18.0) on a Macintosh workstation (macOS Ventura, version 13.4.1). Graphs were created using GraphPad Prism (version 10.1.1).

## Data Availability

The datasets generated and/or analyzed during the current study are not publicly available due to data safety regulations but are available from the corresponding author on reasonable request.

## Code availability

The code used in this study is available from the corresponding author upon reasonable request.
